# Clinical characteristics and degree of cardiovascular risk factor control in patients with newly-diagnosed type 2 diabetes in Catalonia

**DOI:** 10.3389/fendo.2024.1339879

**Published:** 2024-02-08

**Authors:** Anna Ramírez-Morros, Josep Franch-Nadal, Jordi Real, Queralt Miró-Catalina, Magdalena Bundó, Bogdan Vlacho, Didac Mauricio

**Affiliations:** ^1^ Grup de Recerca Epidemiològica en Diabetes des de l’Atenció Primària (DAP-CAT) Group, Unitat de Suport a la Recerca Barcelona, Fundació Institut Universitari per a la recerca a l’Atenció Primària de Salut Jordi Gol i Gurina (IDIAPJGol), Barcelona, Spain; ^2^ Gerència Territorial de la Catalunya Central, Institut Català de la Salut, Sant Fruitós de Bages, Spain; ^3^ Center for Biomedical Research on Diabetes and Associated Metabolic Diseases (CIBERDEM), Instituto de Salud Carlos III, Barcelona, Spain; ^4^ Digital Health and Clinical Validation Center for Digital Health Solutions, Hospital de la Santa Creu i Sant Pau, Barcelona, Spain; ^5^ Health Promotion in Rural Areas Research Group, Unitat de Suport a la Recerca de la Catalunya Central, Fundació Institut Universitari per a la recerca a l’Atenció Primària de Salut Jordi Gol i Gurina (IDIAPJGol), Sant Fruitós de Bages, Spain; ^6^ Primary Health Care Center Ronda Prim, Gerència d’Àmbit d’Atenció Primària Metropolitana Nord de Barcelona, Institut Català de la Salut, Mataró, Spain; ^7^ Institut de Recerca Hospital de La Santa Creu I Sant Pau, Barcelona, Spain; ^8^ Department of Endocrinology and Nutrition, Hospital Universitari de la Santa Creu i Sant Pau, Barcelona, Spain; ^9^ Department of Medicine, University of Vic – Central University of Catalonia, Vic, Spain

**Keywords:** sex, gender, type 2 diabetes, newly diagnosis, cardiovascular risk factor

## Abstract

**Introduction:**

Women with type 2 diabetes mellitus (T2DM) face a greater risk of cardiovascular disease (CVD) and encounter challenges in managing cardiovascular risk factors (CVRF); however, limited data are available in individuals with newlydiagnosed T2DM.

**Methods:**

This study aimed to examine differences between women and men at the onset of T2DM in terms of clinical characteristics, glycaemic status, and CVRF management. This was a retrospective cohort study including subjects with newly-diagnosed T2DM from the System for the Development of Research in Primary Care (SIDIAP) database in Catalonia (Spain). Sex differences (Dif) were assessed at baseline and 1-year post-diagnosis, by calculating the absolute difference of means or proportions.

**Results:**

A total of 13,629 subjects with newly-diagnosed T2DM were analyzed. Women were older and had a higher BMI than men. At baseline, women had higher total cholesterol [Dif (95%CI) 10 mg/dL (9.1/10.8)] and low-density lipoprotein cholesterol (LDL-c) [Dif (95%CI) 7 mg/dL (6.3/7.7)], while men had higher rates of smoking and alcohol intake. Lipid target achievement was lower in women, in both primary prevention (LDL-c < 100 mg/dL) [Dif (95%CI) -7.3 mg/dL (-10.5/-4.1)] and secondary prevention (LDL-c < 70 mg/dL) [Dif (95%CI) -8.3 mg/dL (-17.3/0.7)], along with lower statin and antiplatelet prescriptions, especially one year after diagnosis. Changes in clinical and laboratory data one year post-diagnosis revealed that, in the primary prevention group, men experienced greater improvements in total cholesterol, LDL-c and triglycerides, while women had less success in achieving CVRF control targets compared to men. Additionally, cardiovascular events, such as coronary artery disease and peripheral artery disease increased more in men than in women within the first year of diagnosis, especially in primary prevention subjects.

**Conclusion:**

Differences between men and women CVRF are already apparent at the onset of T2DM, particularly in primary prevention, with notable differences in lipid profile and target level attainment.

## 1 Introduction

The global estimation of diabetes among adults aged 20-79 years is projected to increase from 536.6 million people in 2021 to 783.2 million people in 2045 with a predicted expenditure of USD 1,054 billion, which represents an increase of 9.1% compared to that of 2021 (1). Additionally, diabetes-related mortality in 2021 represented 12.2% of global deaths from all causes in people aged 20-79 years. Diabetes-associated deaths among women are reported to be much higher than in men, especially after the age of 60-70 years (2).

Prospective studies and meta-analysis have shown that women with diabetes have a higher risk of cardiovascular disease (CVD) than men in comparison with their non-diabetic counterparts (3–7), and have greater difficulty in achieving the therapeutic targets of cardiovascular risk factor (CVRF) control, especially lipid control (8–11). An Italian study attempted to establish the precise time at which excess risk begins in women. Their findings revealed that excess risk of acute myocardial infarction and major cardiovascular events started earlier (46 years), and lasted over the age of 85 years, while ‘risk-windows’ started later and had a shorter duration for congestive heart failure (56-65 years) and ischemic stroke (66-75 years) (12).

Most studies have been conducted with prevalent cases of diabetes and have not described the sex differences at the onset of type 2 diabetes mellitus (T2DM). Available data is scarce regarding sex differences in prediabetes, the prelude to diabetes, and in people with newly-diagnosed T2DM. Regarding CVRFs, conversion from prediabetes to diabetes has been shown to be associated with an increased body mass index (BMI), fasting insulin, triglycerides (TGs) and blood pressure (BP), and lower high-density lipoprotein cholesterol (HDL-c), differences that were greater in women than men (13, 14). The Coronary Artery Risk Development in Young Adults (CARDIA) study, a longitudinal observational cohort study done in the US metropolitan communities, found that CVRF worsened more rapidly after the development of T2DM in women than in men, but they did not find differences between women and men before diabetes (15). However, it should be noted that the proportion of those in the cohort who developed diabetes was small. Overall, to our knowledge, no studies have evaluated the differences between men and women in a large cohort at the onset of T2DM. For this reason, this study sought to describe the clinical characteristics, the degree of glycaemic control and cardiovascular risk factor control at the onset on T2DM, together with any changes 1 year post-diagnosis, in a population-based cohort of newly-diagnosed subjects with T2DM in Catalonia (Spain).

## 2 Materials and methods

### 2.1 Study design

This was a retrospective population-based cohort study. The data were sourced from the Information System for the Development of Research in Primary Care (SIDIAP) database, a large and comprehensive clinical database that is available for research purposes using the ECAP software information system (16). The SIDIAP database captures pseudo-anonymized data from electronic medical records pertaining to individuals who are registered with the primary healthcare centres of the *Institut Català de la Salut* (ICS), the largest healthcare provider in Catalonia (Spain), encompassing about 80% of the Catalan population (5.8 million people).

The study was performed using data extracted from the database covering the period of January 1^st^, 2017 to December 31^st^, 2018. We included all subjects with a first diagnosis of T2DM, defined as the presence of the diagnostic ICD-10 (International Classification of Diseases 10) codes E11 and E14, during 2017 and followed up during 2018. To be included, subjects had to have been in the SIDIAP database for at least 365 days prior to the diagnosis of diabetes and be aged over 30 years. The exclusion criteria were a previous diagnosis of any type of diabetes mellitus and previous prescription of glucose-lowering drugs. The cut-off dates for the analysis were at the onset of diabetes and 1 year after the onset of diabetes. To assess the magnitude of change in clinical variables and in CVRF targets only those with baseline and 1-year post diagnosis data were included in these analyses.

The study was approved by the Ethics Committee of the Primary Healthcare University Research Institute (IDIAP) Jordi Gol (P22-207), Barcelona.

### 2.2 Study variables

The variables included in the study were: age, sex, smoking habit, alcohol use (high-risk alcohol use was defined as the consumption of 21 alcohol units/week in men and 14 units/week in women), BMI, blood glucose level, glycated hemoglobin (HbA1c), estimated glomerular filtration rate (eGFR) with the CKD-EPI (Chronic Kidney Disease Epidemiology Collaboration) formula, lipid profile including total cholesterol, high-density lipoprotein cholesterol (HDL-c), low-density lipoprotein cholesterol (LDL-c) and triglycerides (TGs), blood pressure (BP) (diastolic [dBP] and systolic ([sBP]), hypertension and dyslipidaemia (defined by the ICD-10 diagnostic code [hypertension I10-13, I15, dyslipidaemia E78] and/or a record of lipid-lowering or antihypertensive drug treatment, respectively). Chronic kidney disease was defined as eGFR <60mL/min and/or albumin/creatinine ratio >30mg/g according to the Kidney Disease: Improving Global Outcomes (KDIGO) CKD Work Group guidelines (17).

CV risk was measured using the SCORE2-Diabetes, a new algorithm developed to predict 10-year risk of CVD in individuals with T2DM and without prior history of CVD (risk categories are based on age group (5 year) from 40 to 70 years old) (18). For those with previous CVD, diagnostic codes for macrovascular disease were collected, including coronary artery disease (CAD; ICD-10 codes I20-I24), cerebrovascular disease (ICD-10 codes I63, I64, G45, G46) and peripheral artery disease (PAD; ICD-10 code 173.9). New events of these diagnostic codes occurring during the first year after T2DM onset were also collected for all subjects. Variables of glucose-lowering, lipid-lowering, anti-hypertensive and antiplatelet treatments were also included. For antidiabetic treatment, “baseline” was at 3 months after diagnosis to give enough time for the establishment of prescribed drugs in the first instance. Targets for CVRF control were established in accordance with the European Society of Cardiology (ESC) guidelines (HbA1c < 7%, BP < 140/85 mmHg, LDL-c < 100 mg/dL for those at high CV risk and LDL-c < 70mg/dL for those at very high CV risk) (19).

### 2.3 Statistical analysis

Continuous variables were expressed by mean and standard deviation and categorical variables as frequency and percentage. To evaluate the association between clinical variables and sex, the absolute difference between women and men in the means or proportions (Dif) and their 95% confidence interval (95% CI) were calculated. To evaluate changes from baseline to 1-year after, we estimated the percentage difference ((final value-initial value)/initial value*100) for each individual for each of the continuous variables, and we described the values with mean and standard deviation according to sex. To assess the normality of continuous variables, this study used skewness and kurtosis (20–22). Typically, an absolute skewness value greater than 3 and a kurtosis value greater than 10 may indicate a potential issue with normality. West et al. (23) suggested that the absolute value of skewness and kurtosis should not be greater than 2 and 7. The t-test contrast was used to assess whether there were differences in the percentile changes between sexes. For the categorical variables, the number and percentage of subjects who improved their CVRF control was calculated. We used the chi-square test to analyze if there were differences between sexes since all expected frequencies were higher than 5. An improvement of CVRF was assumed if targets were not achieved at baseline but were achieved 1-year post-diagnosis. A p-value <0.05 was considered to be statistically significant and all contrasts were two-tailed. All analyses were performed using R free software environment for statistical computing (v3.5.1).

## 3 Results

### 3.1 Baseline characteristics

**Figure 1:**
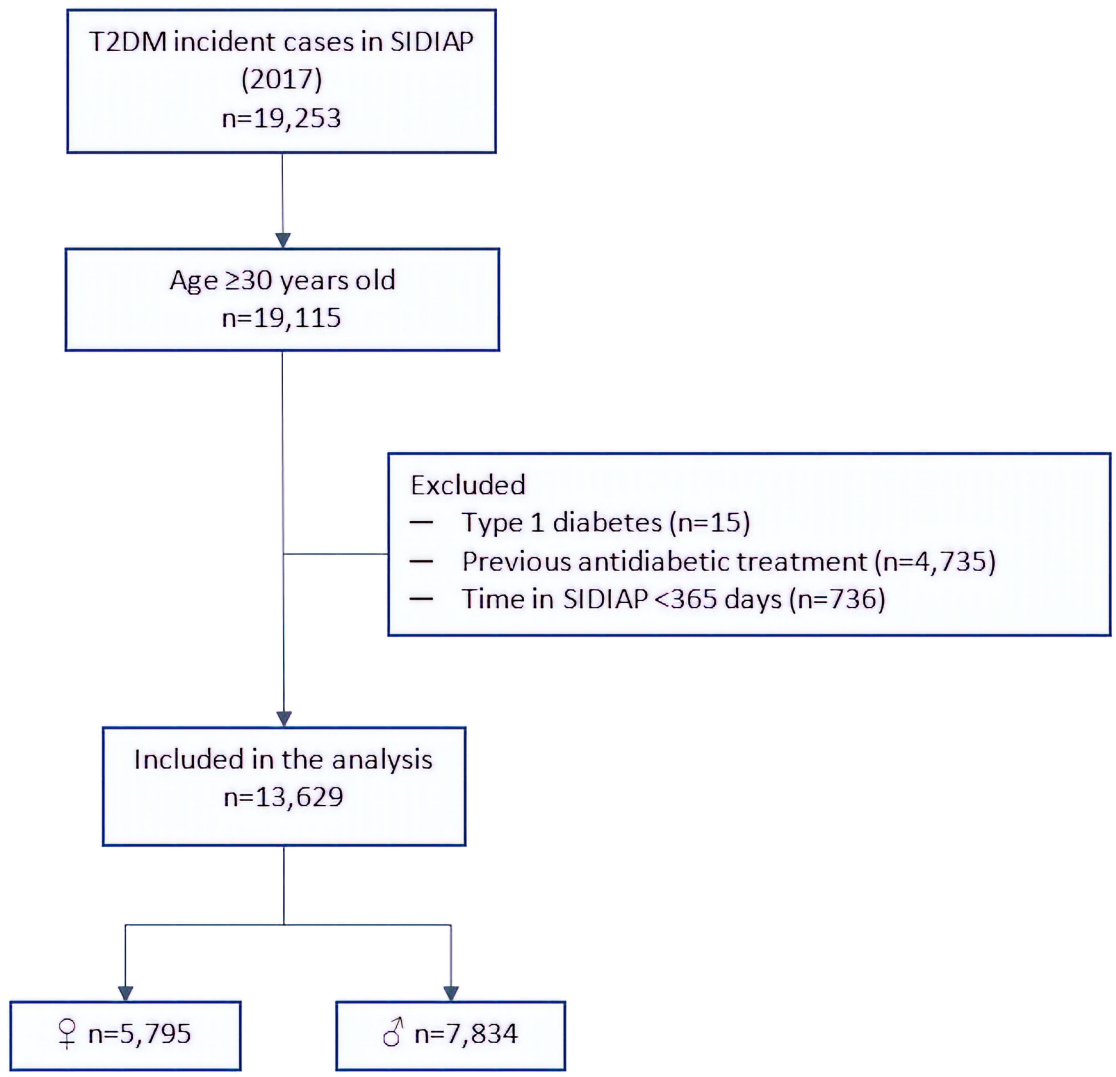
Flow chart of the sample selection. T2DM, type 2 diabetes mellitus; SIDIAP, Information System for the Development of Research in Primary Care.

A total of 19,253 incident cases of T2DM were identified in the SIDIAP database, of which 13,629 subjects (5,795 women and 7,834 men) were included in the study i.e. subjects with a new diagnosis of T2DM, ≥30 years old, with at least 1 year of data in the SIDIAP database (**Figure 1**).

The baseline characteristics and sex differences of the population are shown in **Table 1**. The mean age was 63.4 ± 13.2 years, with a majority in the middle-aged and older age groups. Women had a higher mean age at the onset of T2DM compared to men (65.8 vs. 63.4 years), with a notable difference in the proportion of subjects over 75 years diagnosed with T2DM (26.2% vs 16.0%). Overall, 21.8% of subjects were smokers, while just over half were teetotallers. Smoking and alcohol consumption were significantly higher in men than in women. Nearly twice as many men were smokers or former smokers (65.7% vs. 27.7%) compared to women, while women were more likely to be teetotallers (76.9% vs. 42.6%). Most individuals were either overweight (33.1%) or obese (58.7%). Women had a mean BMI 1.4 points higher than men (32.6 kg/m^2^ vs. 31.2 kg/m^2^), with the greatest difference in the proportion of subjects in the ≥35 kg/m^2^ range (31.1% vs 20.1%).

**Table 1:** Baseline characteristics of incident T2DM.

Variable	n	Total	n	Women	n	Men	Dif (95%CI)
Age (years), mean ± SD	13,629	63.4 ± 13.2	5,795	65.8 ± 13.5	7,834	61.7 ± 12.8	4.1 (3.9/4.3)
Age group (years), n (%)	13,629		5,795		7,834		
30 - <50		2,279 (16.7)		774 (13.4)		1505 (19.2)	-5.8 (-7.1/-4.6)
50 - <65		5,164 (37.9)		1,934 (33.4)		3,230 (41.2)	-7.8 (-9.7/-6.0)
65 - <75		3,418 (25.1)		1,571 (27.1)		1,847 (23.6)	3.5 (1.9/5.1)
≥75		2,768 (20.3)		1,516 (26.2)		1,252 (16.0)	10.2 (8.7/11.7)
Smoking habit, n (%)	12,751		5,470		7,281		
Smoker		2,780 (21.8)		796 (14.6)		1,954 (27.3)	-12.7 (-14.2/-11.2)
Former smoker		3,515 (27.6)		718 (13.1)		2,797 (38.4)	-25.3 (-26.8/-23.8)
Alcohol use, n (%)	7,736		3,333		4,403		
Teetotal		4,436 (57.3)		2,562 (76.9)		1,874 (42.6)	34.3 (31.8/36.8)
Low Risk		3,017 (39.0)		745 (22.4)		2,272 (51.6)	-29.3 (-31.8/-26.7)
High Risk		283 (3.7)		26 (0.8)		257 (5.8)	-5.1 (-5.6/-4.5)
BMI (kg/m^2^), mean ± SD	7,666	31.8 ± 5.5	3,385	32.6 ± 6.0	4,281	31.2 ± 5.1	1.4 (1.3/1.6)
BMI range (kg/m^2^), n (%)	7,666		3,385		4,281		
<25		629 (8.2)		266 (7.9)		363 (8.5)	-0.6 (-1.9/0.6)
25-<30		2,540 (33.1)		962 (28.4)		1,578 (36.9)	-8.4 (-11.0/-5.9)
30-<35		2,584 (33.7)		1,104 (32.6)		1,480 (34.6)	-2.0 (-4.6/0.7)
≥35		1,913 (25.0)		1,053 (31.1)		860 (20.1)	11.0 (8.7/13.3)
Glucose (mg/dL), mean ± SD	11,359	153.5 ± 54.3	5,001	146.8 ± 46.8	6,358	158.7 ± 59.1	-11.9 (-12.8/-10.9)
HbA1c (%), mean ± SD	8,150	7.1 ± 1.5	3,620	6.9 ± 1.2	4,530	7.3 ± 1.6	-0.3 (-0.4/-0.3)
Lipids							
Total-c (mg/dL), mean ± SD	11,022	207.3 ± 44.8	4,880	212.8 ± 42.1	6,142	202.8 ± 46.3	10.0 (9.1/10.8)
HDL-c (mg/dL), mean ± SD	9,960	48.8 ± 12.5	4,401	52.8 ± 12.7	5,559	45.5 ± 11.4	7.3 (7.1/7.6)
LDL-c (mg/dL), mean ± SD	8,981	123.9 ± 35.1	4,108	127.7 ± 34.8	4,873	120.7 ± 35.0	7.0 (6.3/7.7)
TGs (mg/dL), mean ± SD	10,209	195.4 ± 165.6	4,495	175.2 ± 118.1	5,714	211.3 ± 193.5	-36.1 (-39.1/-33.1)
Dyslipidemia, n (%)	13,629	7,090 (52.0)	5,795	3,156 (54.5)	7,834	3,934 (50.2)	4.2 (2.3/6.1)
Blood pressure							
sBP (mmHg), mean ± SD	10,206	134.4 ± 15.3	4,449	133.4 ± 15.3	5,757	135.2 ± 15.2	-1.8 (-2.1/-1.5)
dBP (mmHg), mean ± SD	10,206	79.0 ± 10.7	4,449	78.0 ± 10.3	5,757	79.8 ± 10.9	-1.9 (-2.1/-1.7)
Hypertension, n (%)	13,629	8,480 (62.2)	5,795	3,760 (64.9)	7,834	4,720 (60.3)	4.6 (2.8/6.5)
Chronic kidney disease, n (%)	11,282	1,845 (16.4)	4,978	869 (17.5)	6,304	976 (15.5)	2.0 (0.5/3.4)
eGFR (mL/min), mean ± SD	11,243	79.2 ± 14.9	4,963	78.0 ± 15.7	6,280	80.2 ± 14.2	-2.2 (-2.5/-1.9)
Score2-Diabetes (%), mean ± SD	4,369	11.2 ± 5.2	2,004	9.6 ± 4.2	2,365	12.6 ± 5.5	-3.0 (-3.2/-2.9)
Age 40-<45 years	211	6.2 ± 4.2	83	4.1 ± 2.8	128	7.5 ± 4.4	-3.4 (-3.5/-3.3)
Age 45-<50 years	410	7.9 ± 5.2	144	5.5 ± 3.6	266	9.2 ± 5.5	-3.7 (-3.9/-3.6)
Age 50-<55 years	537	8.9 ± 5.6	217	6.6 ± 3.8	320	10.5 ± 6.0	-3.8 (-4.0/-3.7)
Age 55-<59 years	656	9.4 ± 4.3	270	7.4 ± 3.3	386	10.8 ± 4.3	-3.4 (-3.5/-3.2)
Age 60-<64 years	713	10.9 ± 4.2	295	8.7 ± 2.9	418	12.5 ± 4.3	-3.8 (-4.0/-3.7)
Age 65-<69 years	785	12.0 ± 3.9	394	9.9 ± 2.5	391	14.1 ± 4.0	-4.1 (-4.2/-4.0)
Macrovascular disease, n (%)	13,629	2,087 (15.3)	5,795	636 (11.0)	7,834	1,451 (18.5)	-7.5 (-8.7/-6.4)
CAD	2,087	1,168 (56.0)	636	295 (46.4)	1,451	873 (60.2)	-13.8 (-20.9/-6.7)
Cerebrovascular disease	2,087	799 (38.3)	636	336 (52.8)	1,451	463 (31.9)	20.9 (14.1/27.7)
PAD	2,087	383 (18.4)	636	65 (10.2)	1,451	318 (21.9)	-11.7 (-16.4/-7.0)

Dif, difference between groups; SD, standard deviation; BMI, body mass index; HbA1c, glycated hemoglobin; Total-c, total cholesterol; HDL-c, high density lipoprotein cholesterol; LDL-c, low density lipoprotein cholesterol; TGs, triglycerides; sBP, systolic blood pressure; dBP, diastolic blood pressure; eGFR, estimated glomerular filtration rate; CAD, coronary artery disease; PAD, peripheral artery disease; NIAD, noninsulin antidiabetic drug. All comparisons were significant except for BMI range < 25kg/m^2^, BMI range 30-<35kg/m^2^ and Combined NIAD.

Mean glucose and glycosylated hemoglobin levels were significantly lower in women. Dyslipidaemia was present in 52% of the population, with most lipid profile parameters higher in women except for TGs (total cholesterol, 212.8 vs. 202.8 mg/dL; HDL-c, 52.8 vs. 45.5 mg/dL; LDL-c, 127.7 vs. 120.7 mg/dL). Hypertension was present in 62.2% of individuals; it was slightly more prevalent in women (64.9% vs. 60.3%), although women had a 1.8-point lower mean systolic BP and a 1.9-point lower mean diastolic BP. Overall, the proportion of individuals with chronic kidney disease at the time of T2DM diagnosis was 16.4%, and this was higher in women (17.5%) than in men (15.5%). Mean eGFR, although within normal range, was somewhat lower in women than in men (78.0 mL/min vs 80.2 mL/min). Previous CVD was present in 15.3% of the cohort with a significant predominance in men (18.5% vs 11%). Men were more likely to have CAD (60.2% vs 46.4%) and PAD (21.9% vs 10.2%), while women were more likely to have cerebrovascular disease (52.8% vs 31.9%). As expected, CV risk, measured using the Score2DM, increased with age, particularly in those over 65, but was lower in women across all age ranges (ranging from 4.1 to 9.9 in women and from 7.5 to 14.1 in men).

The baseline characteristics were also analyzed separately for subjects in primary prevention (i.e. subjects without a CVD condition when diagnosed with T2DM) (**Supplementary Table 1**) and for the subjects in secondary prevention (i.e. in subjects with an existing CVD condition at T2DM onset) (**Supplementary Table 2**), showing similar results to the whole population.

### 3.2 Glucose control

The antidiabetic treatments at baseline (i.e 3 months after diagnosis) and at 1-year post-diagnosis are shown in **Table 2**. The majority of subjects did not receive any treatment within 3 months of diagnosis, with 61.4% of women and 56.8% of men not taking any medications. After 1 year of diagnosis, a similar situation persisted, with 57.8% of women and 55.6% of men not taking any medications. Around 33% were prescribed non-insulin antidiabetic drugs (NIAD), increasing up to 35% after 1 year of diagnosis. Other treatments like combined NIAD or insulin were less common but slightly more frequent in men. Changes in prescription patterns were observed over the year following diagnosis, with a decrease in sex differences in medication usage in almost every treatment group.

**Table 2:** Antidiabetic treatment during the first year of T2DM by sex.

	Baseline§						1 year post-diagnosis			
Variable	n	Women	n	Men	Dif (95%CI)	n	Women	n	Men	Dif (95%CI)
Antidiabetic treatment, n (%)	5,795		7,834			5,795		7,834		
No treatment		3,560 (61.4)		4,449 (56.8)	4.6 (2.8/6.5)*		3,349 (57.8)		4,356 (55.6)	2.2 (0.3/4.1)*
NIAD		1,819 (31.4)		2,676 (34.2)	-2.8 (-4.5/-1.0)*		2,072 (35.8)		2,857 (36.5)	-0.7 (-2.5/1.1)
Combined NIAD		191 (3.3)		292 (3.7)	-0.4 (-1.0/0.1)		196 (3.4)		362 (4.6)	-1.2 (-1.8/-0.7)*
NIAD&Insulin		111 (1.9)		222 (2.8)	-0.9 (-1.3/-0.5)*		81 (1.4)		145 (1.9)	-0.5 (-0.8/-0.1)*
Insulin		114 (2.0)		195 (2.5)	-0.5 (-0.9/-0.1)*		97 (1.7)		114 (1.5)	0.2 (-0.2/0.6)

Dif, difference between groups; NIAD, noninsulin antidiabetic drug. §Baseline for antidiabetic treatment is at 3 months after diabetes diagnosis. *Significant comparisons.

### 3.3 Cardiovascular risk factor control

As the management of CV risk factors may differ between primary and secondary prevention, an approach from this perspective was adopted.

#### 3.3.1 Primary prevention

Lipid-lowering and anti-hypertensive drug usage for incident T2DM cases without previous CVD were examined (**Table 3**). For subjects with dyslipidaemia, statins were the primary treatment, mainly in women (51.6% vs. 43.3%), followed by fibrates mainly in men (12.3% vs. 5.9%). After one year, statin use increased in men. Subjects with hypertension were frequently treated with renin-angiotensin-aldosterone system (RAAS) blockers, especially in men (68% vs. 64.7%), while diuretic treatment was more common in women (62.6% vs. 48.6%). After one year, the use of RAAS blockers increased in men, while diuretic use decreased in both groups.

**Table 3:** Pharmacological treatment and cardiovascular risk factor control of incident cases of T2DM in primary prevention by sex.

	Baseline						1 year post-diagnosis			
Variable	n	Women	n	Men	Dif (95%CI)	n	Women	n	Men	Dif (95%CI)
Lipid-lowering treatment, n (%)‡	2,646		2,756			2,816		3,052		
Statins		1,364 (51.5)		1,194 (43.3)	8.2 (4.6/11.9)*		1,560 (55.4)		1,592 (52.2)	3.2 (-0.2/6.7)
Ezetimibe		40 (1.5)		33 (1.2)	0.3 (-0.2/0.9)		44 (1.6)		43 (1.4)	0.2 (-0.4/0.7)
Fibrates		155 (5.9)		338 (12.3)	-6.4 (-8.1/-4.7)*		165 (5.9)		352 (11.5)	-5.6 (-7.2/-4.2)*
Statins & Ezetimibe		31 (1.2)		26 (0.9)	0.3 (-0.2/0.7)		32 (1.1)		27 (0.9)	0.2 (-0.2/0.7)
Anti-hypertensive treatment, n (%)‡	3,193		3,446			3,310		3,712		
RAAS blocker (ACEI or ARBII)		2,065 (64.7)		2,342 (68.0)	-3.3 (-6.2/-0.4)*		2,128 (64.3)		2,562 (69.0)	-4.7 (-7.5/-2.0)*
CCBs		665 (20.8)		734 (21.3)	-0.5 (-2.8/1.9)		629 (19.0)		796 (21.4)	-2.4 (-4.7/-0.2)*
Beta-blockers		701 (22.0)		657 (19.1)	2.9 (0.6/5.2)*		700 (21.1)		702 (18.9)	2.2 (0.0/4.4)*
Diuretic		2,000 (62.6)		1,675 (48.6)	14.0 (10.9/17.1)*		1,875 (56.6)		1,639 (44.2)	12.4 (9.5/15.5)*
RAAS blocker & CCB		512 (16.0)		607 (17.6)	-1.6 (-3.7/0.5)		461 (13.9)		651 (17.5)	-3.6 (-5.6/-1.7)*
RAAS blocker & Diuretic		1,455 (45.6)		1,356 (39.3)	6.3 (3.1/9.3)*		1,358 (41.0)		1,348 (36.3)	4.7 (1.8/7.6)*
Target CVRF achievement, n (%)										
HbA1c < 7%	2,577	1,752 (68.0)	2,743	1,573 (57.4)	10.6 (7.1/14.2)*	2,577	2,089 (81.1)	2,743	2,172 (79.2)	1.9 (-0.8/4.6)
BP < 140/85 mmHg	3,380	1,245 (36.8)	3,770	1,045 (27.7)	9.1 (6.4/11.8)*	3,380	1,425 (42.2)	3,770	1,305 (34.6)	7.6 (4.7/10.4)*
LDL-c < 100 mg/dL	2,491	410 (16.5)	2,507	503 (20.1)	-3.6 (-6.3/-0.9)*	2,491	521 (20.9)	2,507	708 (28.2)	-7.3 (-10.5/-4.1)*
LDL-c < 70 mg/dL	2,491	48 (1.9)	2,507	79 (3.2)	-1.3 (-2.0/-0.4)*	2,491	73 (2.9)	2,507	98 (3.9)	-1.0 (-1.9/-0.0)*

Dif, difference between groups; RAAS, renin-angiotensin-aldosterone system; ACEI, angiotensin converting enzyme inhibitors; ARBII, angiotensin II receptor blockers; CCB, calcium channel blocker; CVRF, cardiovascular risk factors; HbA1c, glycated haemoglobin; BP, blood pressure; LDL cholesterol, low density lipoprotein cholesterol. ‡Lipid-lowering and anti-hypertensive treatment, proportion data are calculated based on those with dyslipidaemia and hypertension respectively. *Significant comparisons.

The proportion of subjects achieving CVRF control targets was assessed in those having laboratory test results for both the baseline and 1-year follow-up periods. Target achievement rates for HbA1c and BP were higher in women, while men had better achievement for LDL-c levels. Over the year, men improved their HbA1c and BP targets, reducing the sex differences. Target LDL-c levels were less frequently achieved in women compared to men, both at baseline and, most remarkably, one year after diagnosis, increasing the sex differences [LDL-c<100mg/dL: baseline, Dif (95%CI) -3.6 (-6.3/-0.9); 1 year, Dif (95%CI) -7.3 (-10.5/-4.1)].

**Figure 2:**
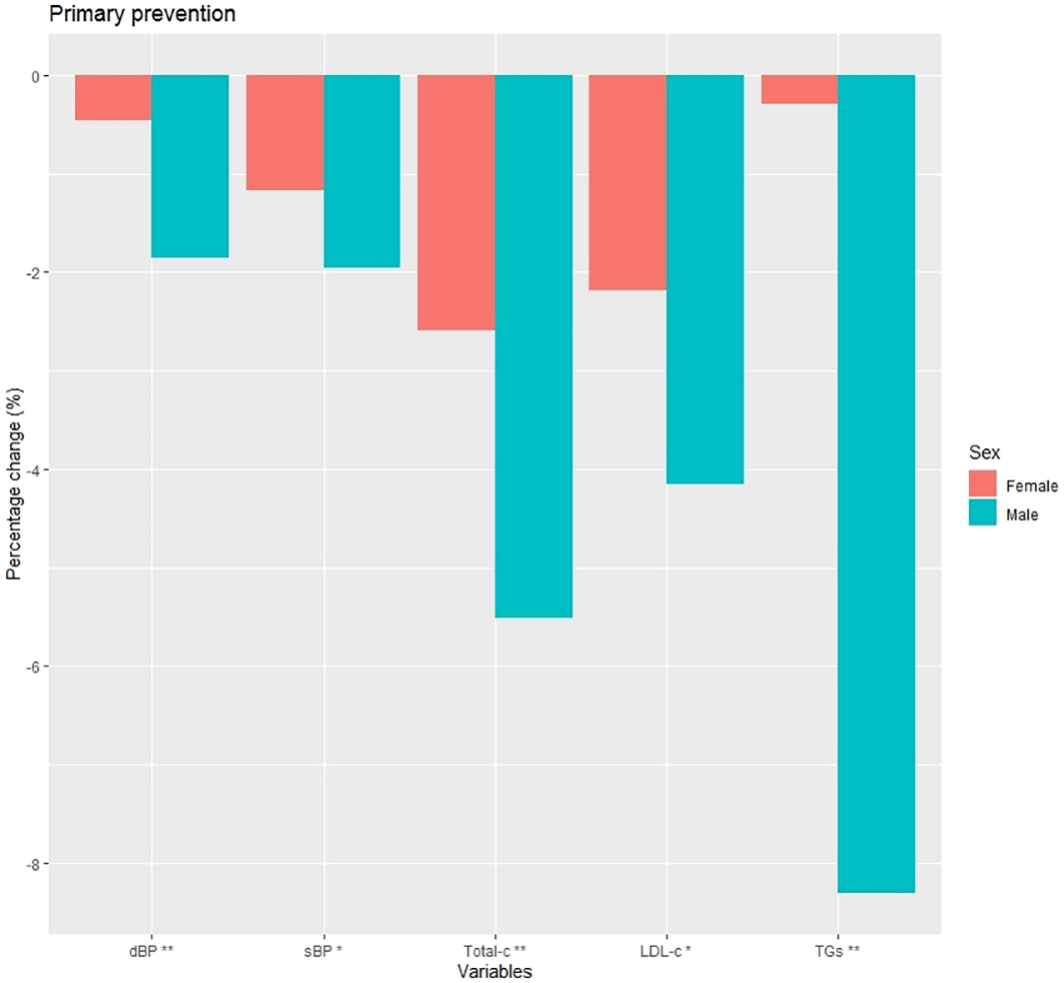
Percentage change at 1 year post-diagnosis in clinical characteristics in primary prevention subjects. dBP (mmHg), diastolic blood pressure; sBP (mmHg), systolic blood pressure; Total-c (mg/dL), total cholesterol; LDL-c (mg/dL), low density lipoprotein cholesterol; TGs (mg/dL), triglycerides; *p<0.01; **p<0.001.

Changes from baseline at 1 year after diagnosis in clinical and laboratory data among women and among men are shown in **Figure 2** and **Supplementary Table 3**. Significant changes from baseline were observed for all parameters. Changes in total cholesterol and LDL-c were more substantial in men, with a greater mean percentage decrease compared to women for both (Total-c: -5.5% vs. -2.6%, p<0.001; LDL-c: -4.2% vs. -2.2%, p=0.004).

**Figure 3:**
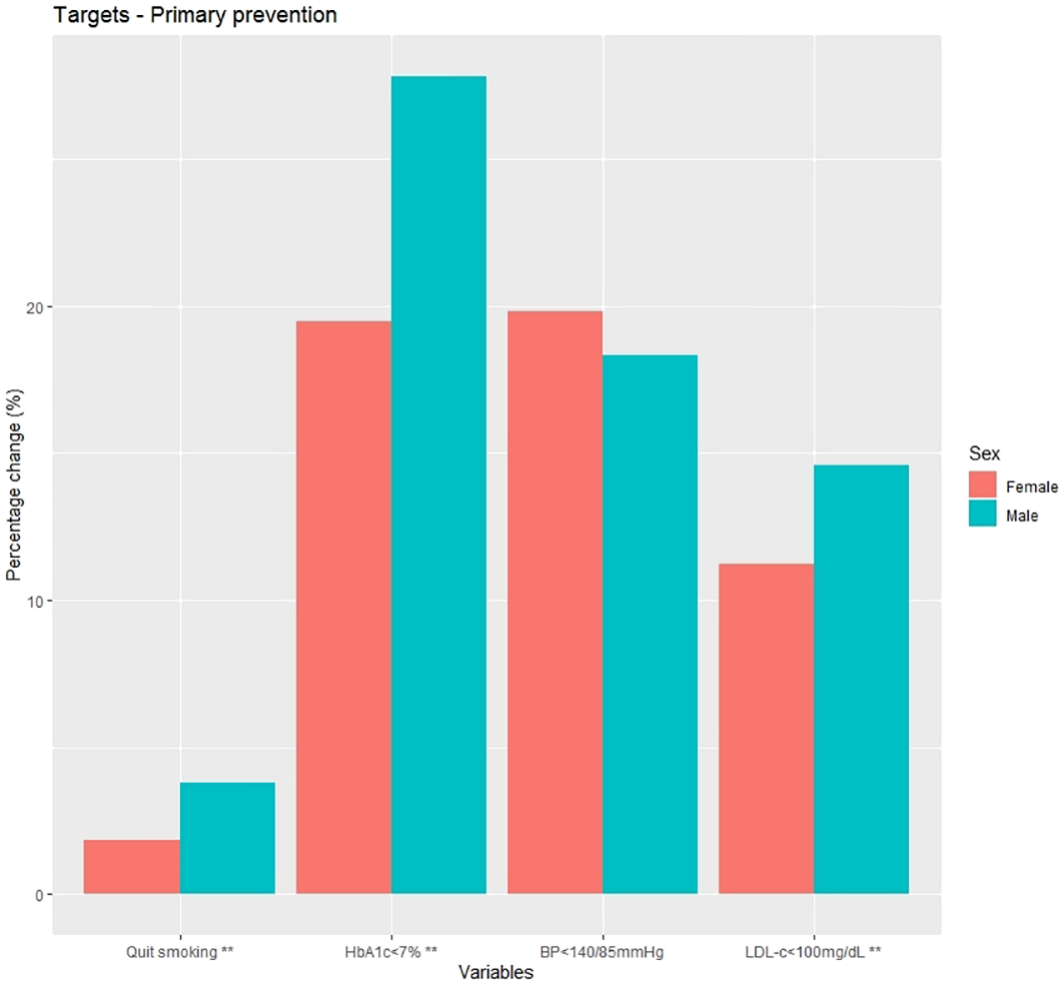
Percentage change at 1 year post-diagnosis in cardiovascular risk factor targets in primary prevention subjects. HbA1c, glycated haemoglobin; BP, blood pressure; LDL-c, low density lipoprotein cholesterol; **p<0.001.

Changes in CVRF targets are shown in **Figure 3** and **Supplementary Table 3**. After 1-year post-diagnosis, women had less success than men in achieving most CVRF targets, including quitting smoking (1.8% vs. 3.8%, p<0.001), improving glycaemic control (19.5% vs. 27.8%, p<0.001), and reaching LDL-c<100mg/dL (11.2% vs. 14.6%, p<0.001).

#### 3.3.2 Secondary prevention

Drug treatments for the control of lipids and BP in those with previous CVD are presented in **Table 4**. Statins and ezetimibe were the most commonly prescribed lipid-lowering treatments. While there were no significant differences in statin use at baseline between men and women, differences were observed 1 year after diagnosis, (78.2% in women vs 84.5% in men; difference: 6.3%). RAAS blockers were the most commonly used BP-lowering treatment in both sexes, with no significant differences at both time points. Diuretics were more frequently prescribed in women than in men, especially at baseline, with an 18.3% difference. Antiplatelet therapy was more frequently prescribed in men than in women, particularly 1 year after diagnosis, with a 10.9% difference.

**Table 4:** Pharmacological treatment and cardiovascular risk factor control of incident cases of T2DM in secondary prevention by sex.

	Baseline						1 year post diagnosis			
Variable	n	Women	n	Men	Dif (95%CI)	n	Women	n	Men	Dif (95%CI)
Lipid-lowering treatment, n (%)‡	510		1,178			522		1,218		
Statins		422 (82.7)		1,025 (87.0)	-4.3 (-9.4/0.9)		408 (78.2)		1,029 (84.5)	-6.3 (-12.0/-0.7)*
Ezetimibe		36 (7.1)		140 (11.9)	-4.8 (-8.8/-0.9)*		42 (8.0)		145 (11.9)	-3.9 (-7.9/0.2)
Fibrates		10 (2.0)		79 (6.7)	-4.7 (-7.0/-2.5)*		13 (2.5)		71 (5.8)	-3.3 (-5.6/-1.1)*
Statins & Ezetimibe		34 (6.7)		133 (11.3)	-4.6 (-8.5/-0.8)*		40 (7.7)		135 (11.1)	-3.4 (-7.2/0.4)
Anti-hypertensive treatment, n (%)‡	567		1,274			575		1,307		
RAAS blocker (ACEI or ARBII)		386 (68.1)		893 (70.1)	-2.0 (-9.1/5.0)		353 (61.4)		841 (64.3)	-2.9 (-10.4/4.5)
CCBs		166 (29.3)		383 (30.1)	-0.8 (-7.8/6.2)		149 (25.9)		348 (26.6)	-0.7 (-7.2/5.8)
Beta-blockers		266 (46.9)		675 (53.0)	-6.1 (-14.0/1.9)		264 (45.9)		685 (52.4)	-6.5 (-14.3/1.3)
Diuretic		378 (66.7)		616 (48.4)	18.3 (10.6/26.0)*		339 (59.0)		571 (43.7)	15.3 (7.6/23.0)*
RAAS blocker & CCB		134 (23.6)		297 (23.3)	0.3 (-5.9/6.6)		114 (19.8)		258 (19.7)	0.1 (-5.5/5.7)
RAAS blocker & Diuretic		295 (52.0)		503 (39.5)	12.5 (4.8/20.3)*		243 (42.3)		428 (32.7)	9.6 (2.2/16.9)*
Antiplatelet therapy, n (%)	636	393 (61.8)	1,451	1,011 (69.7)	-7.9 (-14.52/-1.26)*	636	361 (56.8)	1,451	981 (67.6)	-10.8 (-17.7/-4.0)*
Target CVRF achievement, n (%)										
HbA1c < 7%	302	203 (67.2)	633	421 (66.5)	0.7 (-11.5/12.9)	302	232 (76.8)	633	505 (79.8)	-3.0 (-12.7/6.8)
BP < 140/85 mmHg	467	227 (48.6)	1,029	443 (43.1)	5.5 (-3.6/14.8)	467	259 (55.5)	1,029	545 (53.0)	2.5 (-6.8/11.8)
LDL-c < 70 mg/dL	293	39 (13.3)	626	99 (15.8)	-2.5 (-10.6/5.6)	293	37 (12.6)	626	131 (20.9)	-8.3 (-17.3/0.7)

Dif, difference between groups; RAAS, renin-angiotensin-aldosterone system; ACEI, angiotensin converting enzyme inhibitors; ARBII, angiotensin II receptor blockers; CCB, calcium channel blocker; CVRF, cardiovascular risk factor; HbA1c, glycated haemoglobin; BP, blood pressure; LDL cholesterol, low density lipoprotein cholesterol. ‡Lipid-lowering and anti-hypertensive treatment, proportion data are calculated based on those with dyslipidaemia and hypertension respectively. *Significant comparisons.

In the assessment of subjects achieving HbA1c, BP, and lipid targets at baseline and 1-year post-diagnosis (for those having the lab test value in both periods), no statistically significant differences were found, although men had a notable better achievement of LDL-c<70 mg/dL, particularly 1-year post-diagnosis (difference of -8.3%), compared to women.

**Figure 4:**
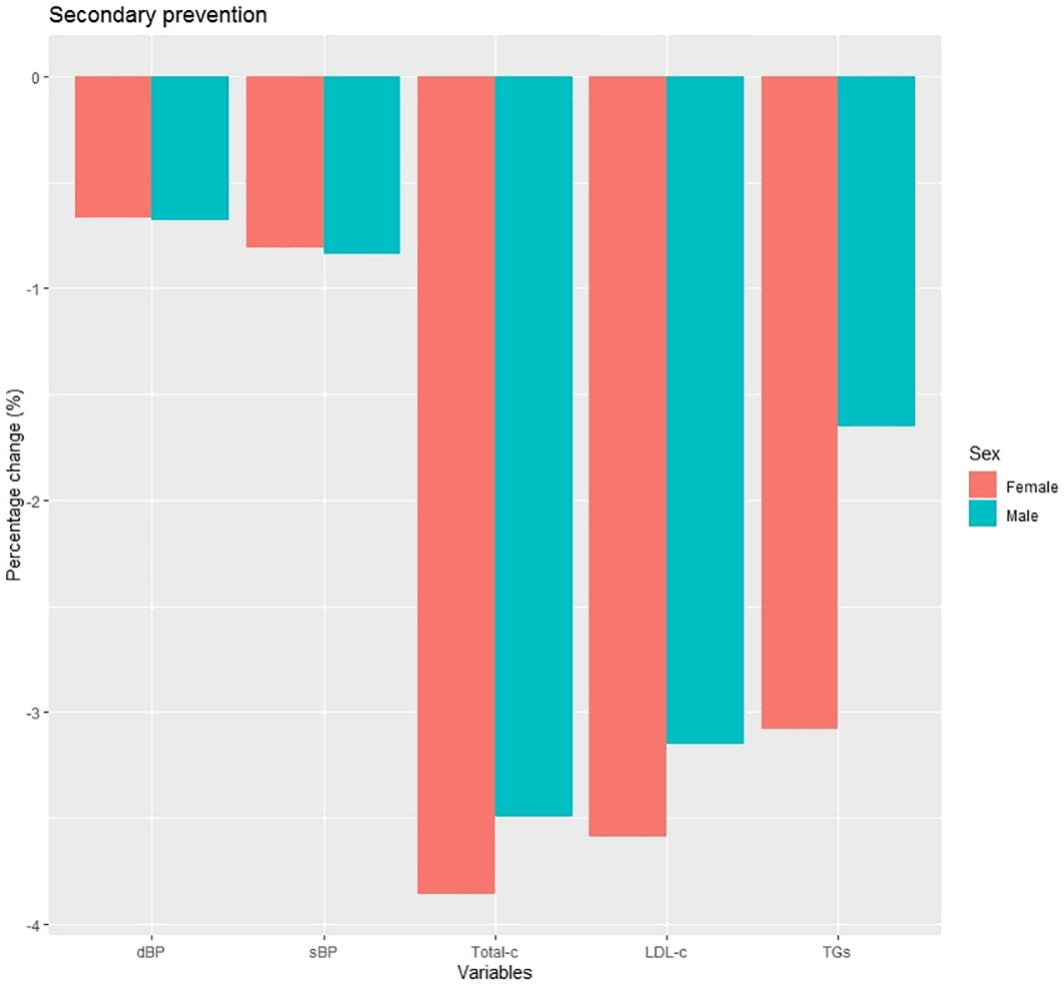
Percentage change at 1 year post-diagnosis in clinical characteristics in secondary prevention subjects. dBP (mmHg), diastolic blood pressure; sBP (mmHg), systolic blood pressure; Total-c (mg/dL), total cholesterol; LDL-c (mg/dL), low density lipoprotein cholesterol; TGs (mg/dL), triglycerides.

**Figure 5:**
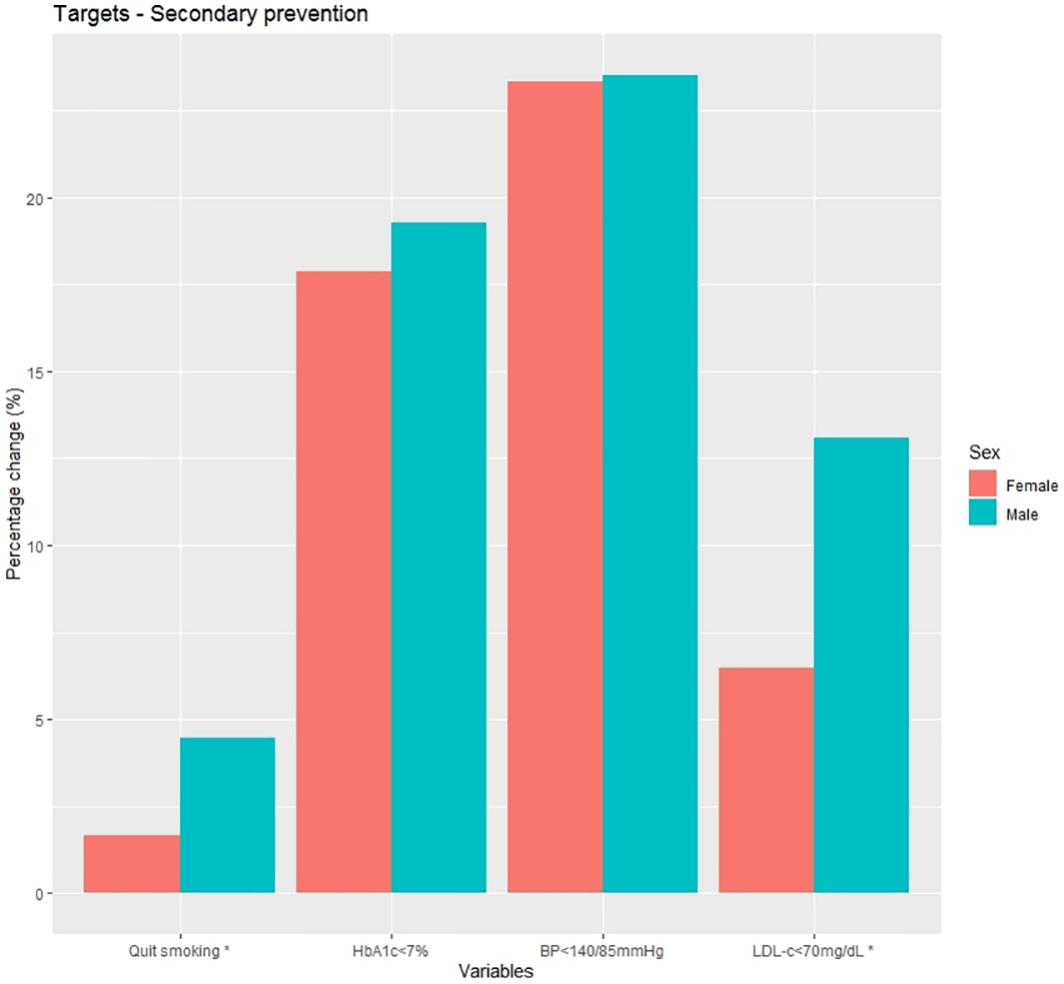
Percentage change at 1 year post-diagnosis in cardiovascular risk factor targets in secondary prevention subjects. HbA1c, glycated haemoglobin; BP, blood pressure; LDL-c, low density lipoprotein cholesterol; *p<0.01.

Changes from baseline at 1 year after diagnosis in clinical and laboratory data are shown in **Supplementary Table 4** and **Figure 4**. The mean percentage change was similar across all variables between the groups, with the most notable change being in glycated hemoglobin, although without statistically significant differences. Changes in CVRF targets are depicted in **Figure 5** and shown in **Supplementary Table 4**. Men showed a greater change in smoking status compared to women (4.5% vs. 1.7%; p=0.002), and the mean percentage change in LDL-c<70 mg/dL was significantly higher in men than in women (13.1% vs. 6.5%; p=0.003).

### 3.4 Cardiovascular events

CV events occurring within the first year of diagnosis were measured (**Table 5**). In primary prevention subjects, events such as CAD and PAD significantly increased in men compared to women (CAD: 1% vs. 0.6%, p=0.027; PAD: 0.7% vs. 0.4%, p=0.012). In secondary prevention subjects, the most common events were CAD (5.4% in men and 4.7% in women; p=0.492) in both men and women. No statistically significant differences were observed between sexes for any of the CV events.

**Table 5:** Cardiovascular events at first year of diagnosis of incident T2DM by sex.

Variable	n	Women	n	Men	p-value
Primary prevention	5,159		6,383		
Events of CAD, n (%)		33 (0.6)		65 (1.0)	0.027
Events of cerebrovascular disease, n (%)		44 (0.9)		51 (0.8)	0.750
Events of PAD, n (%)		19 (0.4)		46 (0.7)	0.012
Secondary prevention	636		1,451		
Events of CAD, n (%)		30 (4.7)		79 (5.4)	0.492
Events of cerebrovascular disease, n (%)		12 (1.9)		34 (2.3)	0.513
Events of PAD, n (%)		6 (0.9)		19 (1.3)	0.479

CAD, coronary artery disease; PAD, peripheral artery disease.

## 4 Discussion

This study describes the clinical characteristics, degree of control of CVRFs and their change 1 year after diagnosis according to sex in a large population-based cohort of 13,629 subjects with newly-diagnosed T2DM in Catalonia (Spain). To our knowledge, this is the only large population-based study addressing this topic in southern Europe. Only a few studies have included newly diagnosed T2DM as the primary study population (24–26), and far fewer have done so differentiating by sex (27–29) particularly with the main objective of describing men and women at onset of T2DM (15, 30).

Of the total number of subjects with newly-diagnosed T2DM, the proportion of men was higher than that of women. Women were older than men, evenly distributed in the age ranges from 50 years onwards. The distribution among men was higher in the age range 50-65 years. In agreement with our results, the incidence of T2DM has been reported to be higher in men than in women and in the oldest age groups according to studies in Europe (31, 32) and Spain (33). In secondary prevention subjects, the age at the onset of diabetes was higher, which is evident because CVD develops at older ages (34).

Men were more frequently smokers and former smokers than women, especially in secondary prevention. The role of smoking in CV morbidity and mortality is widely known (35–37) but it has also been associated with an increased risk of T2DM. The pooled relative risk (RR) of T2DM has been reported to be 1.37 for current smoking and 1.14 for former smoking (38).

Several sex differences in baseline characteristics were observed. We found a higher BMI in women at time of diagnosis. It has been estimated that women have a BMI 1.8 kg/m^2^ higher than men at T2DM diagnosis despite similar levels of HbA1c (39). This variance has been primarily linked to sex-specific physiological differences in fat distribution. Notably, women exhibit a distinctive fat distribution characterized by a higher proportion of subcutaneous fat mass and comparatively lower levels of liver and visceral fat content. This favorable pattern changes after post-menopause, when the fat distribution in women transition from a gynoid pattern to an android pattern accompanied by an increase in cardiometabolic risk (40). In parallel, women also tend to display heightened glucose sensitivity in comparison to men (41). A possible consequence of these sex-specific metabolic nuances is that women require a greater weight gain and adiposity accumulation to meet the diagnostic criteria for T2DM. This phenomenon contributes to an extended duration of the prediabetes state in women, where an elevated presence of CV risk factors is evident (42).

In accordance, we observed a poorer lipid profile in women than in men, especially in total cholesterol and LDL-c but not in HDL and TGs. Several studies report similar results in baseline characteristics of prediabetes or newly-diagnosed T2DM subjects (28, 43, 44), and emphasize the more adverse changes in cardiometabolic risk factors in women as a continuous process in the transition from normoglycemia to diabetes (45, 46). Women could potentially face prolonged exposure to hyperglycemia or an inadequate state of glucose levels, leading to heightened vascular damage and increases in CVRFs (47–49). Otherwise, women had lower mean glucose and HbA1c levels than men which may suggest a better insulin sensitivity pattern in women especially before developing TDM2 (50, 51). This is consistent with the baseline characteristics in different studies with prediabetes or newly-diagnosed T2DM subjects (27, 28, 44, 52, 53). In line with this, the DECODE Study group found that impaired fasting glucose was more common in men whereas impaired glucose tolerance was higher in women. As a consequence, diabetes may go undiagnosed, especially in females, as the fasting glucose determination alone is the standard method for diagnosis (54).

Regarding the control of CVRF and its management, differences were observed between sexes, most notably in primary prevention subjects. The achievement of HbA1c < 7% was higher in women than in men, both at baseline and after 1 year, but the differences between the sexes were higher at baseline than thereafter. A higher proportion of women did not receive antidiabetic treatment at baseline, although after 1 year, the prescription of the antidiabetic drugs was similar between groups. Similarly, BP < 140/90 mmHg was better achieved in women than in men and there were no major differences in anti-hypertensive treatment with the exception of diuretics, which were mostly prescribed in women. The use of thiazide diuretics has been associated with an increased risk of developing T2DM as opposed to RAAS blocker use that seems to reduce the risk of T2DM (55).

By contrast, the attainment of LDL-c < 100 mg/dL was worse in women at baseline and even worse 1 year after diagnosis. Statins were more frequently prescribed in women, but the frequency of statin prescription in men 1 year after diagnosis was considerably higher. It should be noted that statins have been reported to increase the risk of T2DM. Factors associated with this effect of statins are the type of statin, the dose and the potency (56).

It is noteworthy that when the CVRF achievement is better in women than men at baseline (e.g. as seen for HbA1c and BP targets), the difference between the sexes narrowed considerably one year after diagnosis, accompanied by a greater intensification of treatment in men. In contrast, the achievement of LDL-c target levels was better for men at baseline and even more so 1 year post-diagnosis i.e. the difference did not narrow over time for women. This fact can be seen in more detail in the percentage change in CVRF targets and also in the percentage change of clinical characteristics where the improvement over time was higher in men than in women (**Supplementary Table 3** and **4**). The reasons why women are not seeing the same improvements as men are not known, however this issue suggests that more aggressive treatment would be useful in women especially in primary prevention (57, 58).

The present study has some limitations. Ethnicity was not known, and we could not differentiate the sample according to this variable. Some residual confounders were not available in our study, such as physical activity, nutritional status, sex hormones or the use of hormone replacement therapy, which appear to play a protective role in the onset of diabetes (59); all these factors together with the socioeconomic status may have yielded somewhat different results. Observational studies and cross-sectional design do not allow the establishment of causal relationships between the variables. The retrospective design of our study introduces the possibility of selection bias, as the study draws on pre-existing records rather than a prospectively designed protocol. Also, there were no data on the doses of the prescribed drugs, on contraindications, or on treatment adherence, which may influence differences in the disease management. Moreover, the SIDIAP database may have limitations related to the accuracy and completeness of the recorded data. Lastly, the dates of the drug prescription and the dates of the blood tests were unknown, and therefore it was not possible to know which came first; however, this issue affected both groups. For future research, a controlled study design, including prospective data collection, could overcome many of the above-mentioned limitations.

In conclusion, this study shows that there are differences between men and women in CV risk factors and their control, not only long after their diagnosis, but also at the onset of the disease, especially in primary prevention. These differences are especially evident in the lipid profile and the achievement of its targets. It is also important to note that improvements in the control of CV risk factors over time (1-year post-diagnosis) were more evident in men than in women, suggesting that women might benefit from a more aggressive treatment approach in the first year after the onset of the disease.

## Data availability statement

Restrictions apply to the availability of some or all data generated or analyzed during this study because they were used under license. Requests to access these datasets should be directed to DM, didacmauricio@gmail.com.

## Ethics statement

The studies involving humans were approved by the Ethics Committee of the Primary Healthcare University Research Institute (IDIAP) Jordi Gol (P22-207). The studies were conducted in accordance with the local legislation and institutional requirements. Written informed consent for participation was not required from the participants or the participants’ legal guardians/next of kin in accordance with the national legislation and institutional requirements.

## Author contributions

AR-M: Data curation, Formal Analysis, Investigation, Methodology, Project administration, Validation, Visualization, Writing – original draft, Writing – review & editing, Conceptualization. JF-N: Conceptualization, Supervision, Validation, Visualization, Writing – review & editing. JR: Formal Analysis, Methodology, Writing – review & editing. QM-C: Formal Analysis, Writing – review & editing. MB: Writing – review & editing, Data curation, Validation, Resources. BV: Writing – review & editing, Data curation, Validation, Resources. DM: Conceptualization, Methodology, Supervision, Validation, Visualization, Writing – review & editing.
