# Supplementary material for: Clinical characteristics and degree of cardiovascular risk factor control in patients with newly-diagnosed type 2 diabetes in Catalonia
Source: Front Endocrinol (Lausanne). 2024 Feb 8;15:1339879. doi: 10.3389/fendo.2024.1339879 (PMC10883380; doi:10.3389/fendo.2024.1339879)
Supplement: Supplementary file 1 [file Table_1.docx]

Supplementary Material

# Supplementary Figures and Tables

Supplementary Table 1 - Dif, difference between groups; SD, standard deviation; BMI, body mass index; HbA1c, glycated hemoglobin; Total-c, total cholesterol; HDL-c, high density lipoprotein cholesterol; LDL-c, low density lipoprotein cholesterol; TGs, triglycerides; sBP, systolic blood pressure; dBP, diastolic blood pressure. All comparisons were significant except for BMI range < 25kg/m^2^ and BMI range 30-<35kg/m^2^.

| Supplementary Table 1 - Baseline characteristics of incident T2DM cases by sex in primary prevention. | | | | | | | | |  |  |
| --- | --- | --- | --- | --- | --- | --- | --- | --- | --- | --- |
|  |  |  |  | |  |  | |  | |  |
|  | n | Women | n | Men | | | Dif (95%CI) | |  |  |
| Age (years), mean ± SD | 5,159 | 64.6 ± 13.3 | 6,383 | 59.9 ± 12.4 | | | 4.7 (4.5/4.9) | |  |  |
| Age group (years), n (%) | 5,159 |  | 6,383 |  | | |  | |  |  |
| 30 - <50 |  | 761 (14.8) |  | 1,446 (22.7) | | | -7.9 (-9.4/-6.4) | |  |  |
| 50 - <65 |  | 1,833 (35.5) |  | 2,761 (43.3) | | | -7.7 (-9.8/-5.7) | |  |  |
| 65 - <75 |  | 1,409 (27.3) |  | 1,411 (22.1) | | | 5.2 (3.5/6.9) | |  |  |
| ≥75 |  | 1,156 (22.4) |  | 765 (12.0) | | | 10.4 (9.0/11.9) | |  |  |
| Smoking habit, n (%) | 4,867 |  | 5,898 |  | | |  | |  |  |
| Smoker |  | 736 (15.1) |  | 1,674 (28.4) | | | -13.3 (-14.9/-11.6) | |  |  |
| Former smoker |  | 635 (13.1) |  | 2,090 (35.4) | | | -22.4 (-24.1/-20.7) | |  |  |
| Alcohol use, n (%) | 2,928 |  | 3,520 |  | | |  | |  |  |
| Teetotal |  | 2,221 (75.9) |  | 1,455 (41.3) | | | 34.5 (31.6/37.4) | |  |  |
| Low Risk |  | 682 (23.3) |  | 1,841 (52.3) | | | -29.0 (-31.9/-26.1) | |  |  |
| High Risk |  | 25 (0.9) |  | 224 (6.4) | | | -5.5 (-6.2/-4.8) | |  |  |
| BMI (kg/m^2^), mean ± SD | 2,990 | 32.7 ± 6.0 | 3,414 | 31.4 ± 5.2 | | | 1.4 (1.2/1.5) | |  |  |
| BMI range (kg/m^2^), n (%) | 2,990 |  | 3,414 |  | | |  | |  |  |
| <25 |  | 234 (7.8) |  | 271 (7.9) | | | -0.1 (-1.5/1.3) | |  |  |
| 25 - <30 |  | 835 (27.9) |  | 1,238 (36.3) | | | -8.3 (-11.3/-5.4) | |  |  |
| 30 - <35 |  | 960 (32.1) |  | 1,168 (34.2) | | | -2.1 (-5.1/0.9) | |  |  |
| ≥35 |  | 961 (32.1) |  | 737 (21.6) | | | 10.6 (7.9/13.2) | |  |  |
| Glucose (mg/dL), mean ± SD | 4,460 | 147.5 ± 47.6 | 5,164 | 161.4 ± 61.0 | | | -13.9 (-15.0/-12.8) | |  |  |
| HbA1c (%), mean ± SD | 3,224 | 6.9 ± 1.2 | 3,695 | 7.3 ± 1.6 | | | -0.4 (-0.4/-0.3) | |  |  |
| Lipids |  |  |  |  | | |  | |  |  |
| Total-c (mg/dL), mean ± SD | 4,349 | 216.0 ± 41.3 | 4,978 | 209.8 ± 44.7 | | | 6.2 (5.4/7.1) | |  |  |
| HDL-c (mg/dL), mean ± SD | 3,911 | 53.0 ± 12.7 | 4,472 | 45.8 ± 11.4 | | | 7.2 (7.0/7.5) | |  |  |
| LDL-c (mg/dL), mean ± SD | 3,666 | 130.5 ± 34.2 | 3,920 | 126.1 ± 33.6 | | | 4.4 (3.6/5.1) | |  |  |
| TGs (mg/dL), mean ± SD | 3,992 | 175.6 ± 119.4 | 4,601 | 218.3 ± 202.9 | | | -42.7 (-46.1/-39.3) | |  |  |
| Dyslipidemia, n (%) | 5,159 | 2,646 (51.3) | 6,383 | 2,756 (43.2) | | | 8.1 (6.0/10.2) | |  |  |
| Blood pressure |  |  |  |  | | |  | |  |  |
| sBP (mmHg), mean ± SD | 3,915 | 133.5 ± 15.3 | 4,563 | 135.9 ± 15.0 | | | -2.4 (-2.7/-2.1) | |  |  |
| dBP (mmHg), mean ± SD | 3,915 | 78.4 ± 10.2 | 4,563 | 81.0 ± 10.5 | | | -2.6 (-2.8/-2.4) | |  |  |
| Hypertension, n (%) | 5,159 | 3,193 (61.9) | 6,383 | 3,446 (54.0) | | | 7.9 (5.8/10.0) | |  |  |
| Chronic kidney disease, n (%) | 4,436 | 680 (15.3) | 5,108 | 658 (12.9) | | | 2.5 (1.0/3.9) | |  |  |

Supplementary Table 2 - Dif, difference between groups; SD, standard deviation; BMI, body mass index; HbA1c, glycated hemoglobin; Total-c, total cholesterol; HDL-c, high density lipoprotein cholesterol; LDL-c, low density lipoprotein cholesterol; TGs, triglycerides; sBP, systolic blood pressure; dBP, diastolic blood pressure. All comparisons were significant except for BMI range < 25kg/m^2^ and BMI range 30-<35kg/m^2^.

| Supplementary Table 2 - Baseline characteristics of incident T2DM cases by sex in secondary prevention. | | | | | |
| --- | --- | --- | --- | --- | --- |
|  | n | Women | n | Men | Dif (95%CI) |
| Age (years), mean ± SD | 636 | 75.3 ± 11.0 | 1,451 | 69.4 ± 11.4 | 5.9 (5.4/6.4) |
| Age group (years), n (%) | 636 |  | 1,451 |  |  |
| 30 - <50 |  | 13 (2.0) |  | 59 (4.1) | -2.1 (-3.7/-0.4) |
| 50 - <65 |  | 101 (15.9) |  | 469 (32.3) | -16.4 (-22.2/-10.6) |
| 65 - <75 |  | 162 (25.5) |  | 436 (30.1) | -4.6 (-10.8/1.6) |
| ≥75 |  | 360 (56.6) |  | 487 (33.6) | 23.0 (16.2/29.9) |
| Smoking habit, n (%) | 603 |  | 1,383 |  |  |
| Smoker |  | 60 (10.0) |  | 310 (22.4) | -12.4 (-17.3/-7.6) |
| Former smoker |  | 83 (13.8) |  | 707 (51.1) | -37.3 (-43.7/-31.1) |
| Alcohol use, n (%) | 405 |  | 883 |  |  |
| Teetotal |  | 341 (84.2) |  | 419 (47.5) | 36.7 (27.7/45.8) |
| Low Risk |  | 63 (15.6) |  | 431 (48.8) | -33.2 (-42.3/-24.2) |
| High Risk |  | 1 (0.3) |  | 33 (3.7) | -3.4 (-4.8/-2.2) |
| BMI (kg/m^2^), mean ± SD | 395 | 31.6 ± 5.4 | 867 | 30.4 ± 4.8 | 1.2 (0.9/1.5) |
| BMI range (kg/m^2^), n (%) | 395 |  | 867 |  |  |
| <25 |  | 127 (32.2) |  | 340 (39.2) | -7.1 (-17.1/2.9) |
| 25 - <30 |  | 144 (36.5) |  | 312 (36.0) | 0.5 (-9.5/10.4) |
| 30 - <35 |  | 92 (23.3) |  | 123 (14.2) | 9.1 (2.2/16.0) |
| ≥35 |  | 32 (8.1) |  | 92 (10.6) | -2.5 (-7.3/2.3) |
| Glucose (mg/dL), mean ± SD | 541 | 141.4 ± 39.6 | 1,194 | 147.1 ± 48.5 | -5.7 (-7.8/-3.6) |
| HbA1c (%), mean ± SD | 396 | 6.9 ± 0.9 | 835 | 7.0 ± 1.3 | -0.1 (-0.2/-0.0) |
| Lipids |  |  |  |  |  |
| Total-c (mg/dL), mean ± SD | 531 | 186.3 ± 38.8 | 1,164 | 173.0 ± 40.7 | 13.3 (11.4/15.1) |
| HDL-c (mg/dL), mean ± SD | 490 | 51.2 ± 12.4 | 1,087 | 44.4 ± 11.3 | 6.8 (6.2/7.4) |
| LDL-c (mg/dL), mean ± SD | 442 | 104.9 ± 31.8 | 953 | 98.6 ± 31.7 | 6.3 (4.6/8.0) |
| TGs (mg/dL), mean ± SD | 503 | 171.6 ± 106.8 | 1,113 | 181.9 ± 145.2 | -10.3 (-16.5/-4.2) |
| Dyslipidemia, n (%) | 636 | 510 (80.2) | 1,451 | 1,178 (81.2) | -1.0 (-6.2/4.2) |
| Blood pressure |  |  |  |  |  |
| sBP (mmHg), mean ± SD | 534 | 132.6 ± 15.5 | 1,194 | 132.6 ± 15.5 | -0.0 (-0.8/0.7) |
| dBP (mmHg), mean ± SD | 534 | 75.0 ± 10.7 | 1,194 | 75.4 ± 11.1 | -0.4 (-0.9/0.1) |
| Hypertension, n (%) | 636 | 567 (89.2) | 1,451 | 1,274 (87.8) | 1.4 (-2.5/5.2) |
| Chronic kidney disease, n (%)† | 542 | 189 (34.9) | 1,196 | 318 (26.6) | 8.3 (1.9/14.6) |

Supplementary Table 3 - SD, standard deviation; dBP, diastolic blood pressure; sBP, systolic blood pressure; Total-c, total cholesterol; LDL-c, low density lipoprotein cholesterol; TGs, triglycerides; HbA1c, glycated hemoglobin; BP, blood pressure. Data are mean of percentage change with the SD and the proportion of change from baseline to 1 year after diagnosis.

| Supplementary Table 3 - Percentage change at 1 year post-diagnosis in clinical characteristics and cardiovascular risk factor targets in primary prevention subjects. | | | | | |
| --- | --- | --- | --- | --- | --- |
| Variable | n | Women | n | Men | p-value |
| Blood pressure, mean ± SD |  |  |  |  |  |
| dBP (mmHg) | 3,380 | -0.5 ± 13.6 | 3,770 | -1.9 ± 12.9 | <0.001 |
| sBP (mmHg) | 3,380 | -1.2 ± 12.1 | 3,770 | -2.0 ± 11.2 | 0.005 |
| Lipids, mean ± SD |  |  |  |  |  |
| Total-c (mg/dL) | 3,284 | -2.6 ± 16.4 | 3,631 | -5.5 ± 17.7 | <0.001 |
| LDL-c (mg/dL) | 2,491 | -2.2 ± 24.3 | 2,507 | -4.2 ± 23.7 | 0.004 |
| TGs (mg/dL) | 2,852 | -0.3 ± 39.7 | 3,201 | -8.3 ± 46.0 | <0.001 |
| Targets, n (%) |  |  |  |  |  |
| Quit smoking | 4,867 | 89 (1.8) | 5,898 | 224 (3.8) | <0.001 |
| HbA1c < 7% | 2,577 | 502 (19.5) | 2,743 | 763 (27.8) | <0.001 |
| BP < 140/85 mmHg | 3,380 | 670 (19.8) | 3,770 | 691 (18.3) | 0.108 |
| LDL-c < 100 mg/dL | 2,491 | 280 (11.2) | 2,507 | 366 (14.6) | <0.001 |

Supplementary Table 4 - SD, Standard Deviation; dBP, diastolic blood pressure; sBP, systolic blood pressure; Total-c, total cholesterol; LDL-c, low density lipoprotein cholesterol; TGs, triglycerides; HbA1c, glycated hemoglobin; BP, blood pressure. Data are mean of percentage change with the SD and the proportion of change from baseline to 1 year after diagnosis.

| Supplementary Table 4 - Percentage change at 1 year post-diagnosis in clinical characteristics and cardiovascular risk factor targets in secondary prevention subjects. | | | | | |
| --- | --- | --- | --- | --- | --- |
| Variable | n | Women | n | Men | p-value |
| Blood pressure, mean ± SD |  |  |  |  |  |
| dBP (mmHg) | 467 | -0.7 ± 16.5 | 1,029 | -0.7 ± 15.2 | 0.998 |
| sBP (mmHg) | 467 | -0.8 ± 14.0 | 1,029 | -0.8 ± 13.0 | 0.970 |
| Lipids, mean ± SD |  |  |  |  |  |
| Total-c (mg/dL) | 409 | -3.9 ± 20.8 | 914 | -3.5 ± 19.3 | 0.761 |
| LDL-c (mg/dL) | 293 | -3.6 ± 23.7 | 626 | -3.2 ± 25.8 | 0.799 |
| TGs (mg/dL) | 366 | -3.1 ± 45.1 | 815 | -1.7 ± 46.3 | 0.619 |
| Targets, n (%) |  |  |  |  |  |
| Quit smoking | 603 | 10 (1.7) | 1,383 | 62 (4.5) | 0.002 |
| HbA1c < 7% | 302 | 54 (17.9) | 633 | 122 (19.3) | 0.610 |
| BP < 140/85 mmHg | 467 | 109 (23.3) | 1,029 | 242 (23.5) | 0.940 |
| LDL-c < 70 mg/dL | 293 | 19 (6.5) | 626 | 82 (13.1) | 0.003 |
